# Learning with few samples in deep learning for image classification, a mini-review

**DOI:** 10.3389/fncom.2022.1075294

**Published:** 2023-01-05

**Authors:** Rujun Zhang, Qifan Liu

**Affiliations:** College of Electronics and Information Engineering, Shenzhen University, Shenzhen, China

**Keywords:** few-shot learning, image classification, deep learning, meta-learning, prior knowledge

## Abstract

Deep learning has achieved enormous success in various computer tasks. The excellent performance depends heavily on adequate training datasets, however, it is difficult to obtain abundant samples in practical applications. Few-shot learning is proposed to address the data limitation problem in the training process, which can perform rapid learning with few samples by utilizing prior knowledge. In this paper, we focus on few-shot classification to conduct a survey about the recent methods. First, we elaborate on the definition of the few-shot classification problem. Then we propose a newly organized taxonomy, discuss the application scenarios in which each method is effective, and compare the pros and cons of different methods. We classify few-shot image classification methods from four perspectives: (i) Data augmentation, which contains sample-level and task-level data augmentation. (ii) Metric-based method, which analyzes both feature embedding and metric function. (iii) Optimization method, which is compared from the aspects of self-learning and mutual learning. (iv) Model-based method, which is discussed from the perspectives of memory-based, rapid adaptation and multi-task learning. Finally, we conduct the conclusion and prospect of this paper.

## 1. Introduction

Deep learning techniques have achieved great success, especially in the field of image processing, such as image classification (Bateni et al., 2022), image registration (Chi et al., 2022), and image segmentation (Gao H. et al., 2022). Traditional deep learning is highly data-dependent, which requires training a lot of data to produce high performance. Besides, data annotation and data collection are time-consuming and labor-intensive in practical application. However, humans have the powerful cognitive ability to learn from a small number of samples. Inspired by this, researchers hope that machine learning can perform rapid modeling by learning a few samples, distinguishing different categories, and identifying new classes like humans. Few-shot learning aims to identify new categories with just a few training samples for each category. A lower amount of training data can significantly reduce the computational cost. Meta-learning, or learning to learn, is the discipline of methodically examining how various machine learning algorithms perform on a variety of learning tasks, then the learned experience or meta-data is used to learn novel tasks much more quickly than other approaches (Vanschoren, 2019).

**Figure 1:**
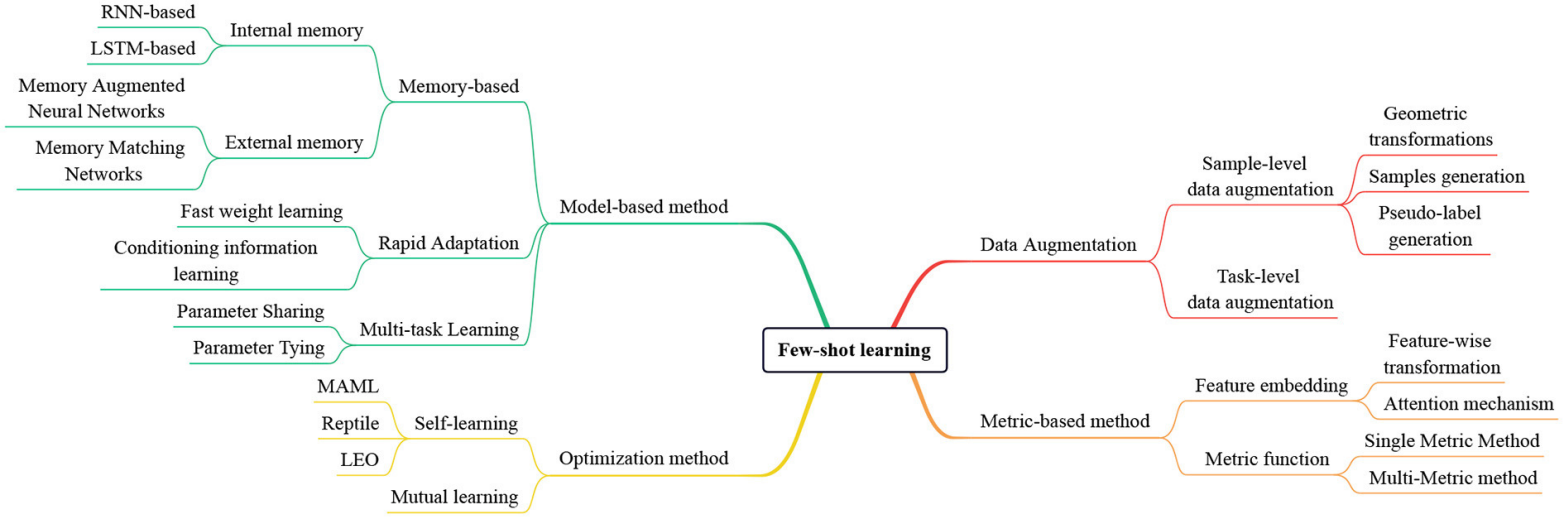
The taxonomy of few-shot learning methods. We divide these methods into four main categories: Data augmentation, metric-based method, optimization method, and model-based method.

The main contribution of our review is a new taxonomic synthesis of recent few-shot learning approaches. The structure of this review is as follows: Section 1 is the introduction; Section 2 is the definition of the few-shot learning problem; Sections 3–6 discuss the four main catogories of few-shot image classification; Section 7 is the applications and challenges of few-shot learning; Section 8 conducts the conclusion and prospect of this paper. The taxonomy of few-shot learning methods is shown in Figure 1.

## 2. The few-shot learning problem definition

**Figure 2:**
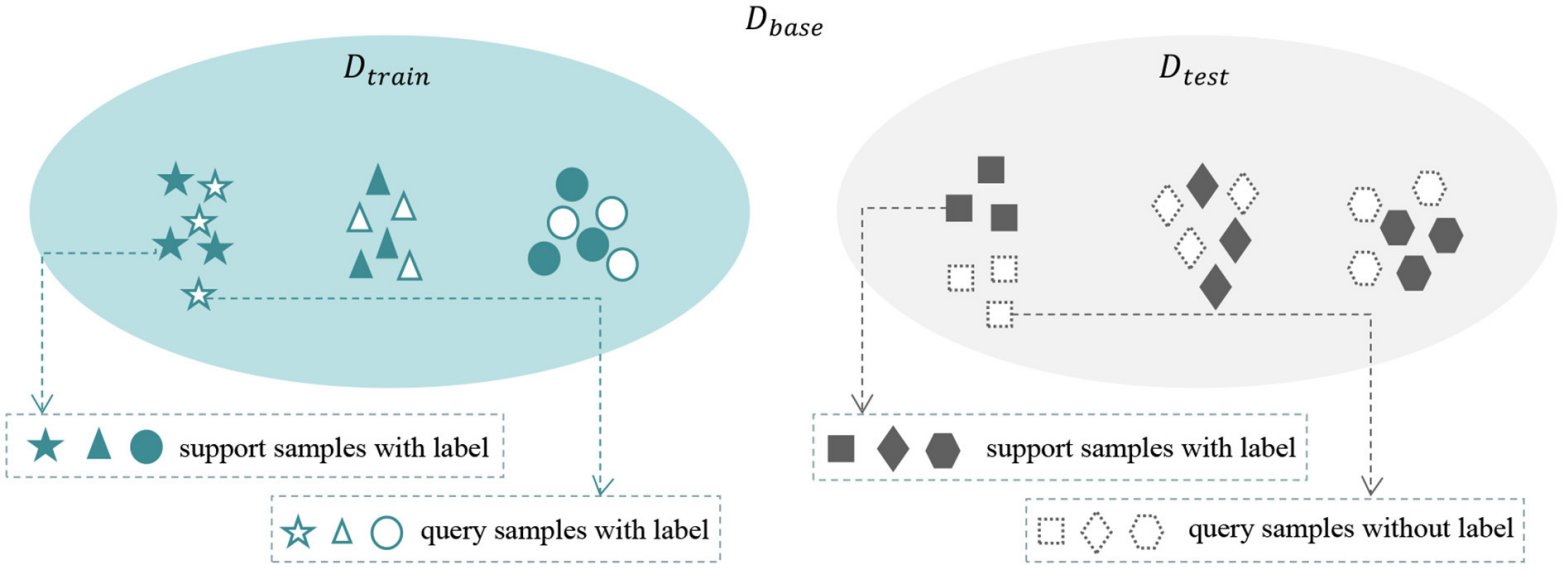
The definition of few-shot learning. The base dataset *D*_*base*_ contains *D*_*train*_ and *D*_*test*_. We train the model with multiple meta-tasks selected from *D*_*train*_ to make it perform good generalization on novel tasks on *D*_*test*_.

We consider a base dataset *D*_*base*_ = (*D*_*train*_, *D*_*test*_), where *D*_*train*_∩*D*_*test*_ = ∅. We randomly select *N* categories and each category with *K* samples from *D*_*train*_ as the support set *S*, the setting is also called the *N*-way *K*-shot problem. Then we select *K*′ samples from the remaining data samples in the same *N* categories as the query set *Q*. It is worth noting that, in the training phase, we construct multiple meta-tasks, each meta-task contains a support set *S* and a query set *Q*. The labels are provided for both support and query samples. In the test phase, the model utilizes the knowledge learned from training phase and a small number of labeled support samples to predict the label of query samples. Few-shot learning aims to train on the seen domain *D*_*train*_ so that the model can quickly adapt to the novel task from the unseen domain *D*_*test*_. The definition of few-shot learning is shown in Figure 2.

## 3. Few-shot learning based on data augmentation

In this section, we mainly focus on sample-level and task-level data augmentation for few-shot image classification. The sample-level few-shot learning utilizes prior knowledge to augment the data, such as using supervised information to enrich the data.

### 3.1. Sample-level data augmentation

Data augmentation is the most important way to solve overfitting: (1) Geometric Transformations, such as scaling (Zhang et al., 2018), rotating (Cai et al., 2022), adding noise to images (Johnander et al., 2022), and transformations (Singh and Mazumder, 2022). However, realizing these augmentation approaches relies on domain knowledge and is labor-intensive (Wang et al., 2020). In addition, these approaches are only applicable to specific datasets and do not have good transfer performance. (2) Samples Generation, which aims at using the existing models to augment the data by generating new samples, such as Generative Adversarial Networks (GAN) (Goodfellow et al., 2020). Subedi et al. (2022) propose the basic data augmentation generative adversarial networks, which uses dual discriminators handling both generated data and generated feature spaces for better learning of the given data. However, using this category of method cannot completely solve the problem of overfitting. (3) Pseudo-Label Generation, which aims to annotate the unlabeled data samples with pseudo-labels (Ding et al., 2021) by exploiting their correlation to the labeled data samples as an auxiliary dataset, then incorporates this dataset into the few-shot learning framework to train the network more effectively with only a few labeled samples. To make pseudo-label for the base dataset, Jian and Torresani (2022) utilize the linear classifier that is trained on the novel classes.

### 3.2. Task-level data augmentation

Compared to sample-level data augmentation, task-level methods are more suitable for some application scenarios. In order to develop generalizable feature representations, Zhang et al. (2021) design the self-supervised learning method to utilize an annotation-free pretext task across all source tasks. Yang (2022) propose a Task-Prior Conditional Variational Auto-Encoder model called TP-VAE to deal with situations where the number of query samples of each class varies from each other. TP-VAE is conditioned on the support set and constrained through a task-level prior regularization.

## 4. Metric-based few-shot image classification

### 4.1. Feature embedding

Feature embedding is used to map samples to feature space for similarity computation. It realizes knowledge transferring from train set to test set. Additionally, it seeks to generalize from a collection of seen domains (train set) to the unseen domain (test set) without using instances from the unseen domain during the training phase and provides better features for subsequent metric-based learning. (1) Feature-wise transformation (Chen Q. et al., 2022) is used to convert feature distributions learned on the source domain into that of target domain. Huang H.-P. et al. (2022) introduce an integrated adaptor module and a feature transformation layer in adaptive vision transformers (ViT), which adapts to different domains with a few samples to achieve robust performance. (2) Attention mechanism can learn some essential features of the target object and improve the learning ability of the model for critical features. Hou et al. (2019) design a novel Cross Attention Network to generate cross attention maps for each pair of class feature and query sample feature. Therefore, it can highlight the target object areas to enhance the feature discriminability for few-shot classification. Chikontwe et al. (2022) clarify a method for few-shot classification, which involves cross-attendance and re-weighting discriminative features. The transformer model is absolutely based on the attention mechanism without any convolutional or recurrent neural network layers. Liu et al. (2020) propose the Universal Representation Transformer (URT) layer, which can effectively learn to convert universal representations into task-adapted representations. Shen and Shuai (2022) exploit contextualization and self-attention capabilities of transition structures. They also utilize a CNN backbone network to better extract generalization features through meta-learning.

### 4.2. Metric function

Metric learning is to learn the metric function for a specific task independently under various tasks. The metric function is designed to compare the similarities between the target category and all source categories in the embedding space for the prediction of the target category (Zhao et al., 2022). For different similarity measures, we can divide these methods into single metric methods and multi-metric methods.

#### 4.2.1. Single metric method

Similarity comparison between support samples and query samples in a single measure space is referred as single metric method. The classic methods contain: Prototype networks (Snell et al., 2017), Graph Neural Networks (Garcia and Bruna, 2017), and Relation networks (Sung et al., 2018). Some recent works have made improvements based on these classical methods. Huang J. et al. (2022) present a series of local-level approaches to improve the few-shot image classification by preventing the discriminative location bias and information loss in local details, which enhance prototypical few-shot learning. Su et al. (2022) propose a few-shot hierarchical classification model using multi-granularity relation networks (HMRN) that takes the inner-class similarity and inter-class relationship into account. This model can improve the ability of classification by reducing the inner-class distance and increasing the inter-class distance. Jia et al. (2022) design a coarse-grained granulation relation network (CGRN) model by denoting the coarse-grained granulation and calculating the similarity of relation score for few-shot classification.

#### 4.2.2. Multi-metric method

It is different from the single metric method, multi-metric method comprehensively compares samples in several different metric spaces to realize prediction of query samples. Employing the multi-metric method to calculate the similarity of samples can reduce the bias of the network to a certain category and increase the robustness of the network. Chen H. et al. (2022) design a fusion module to simultaneously integrate three distinct level similarities: the pixel-level similarity, the similarity of part-level features and global-level features. The query images within a class classified by three distinct level similarity metrics can be more tightly distributed in a smaller feature space, which produce more discriminative feature maps. Gao F. et al. (2022) calculate the distance between the images in multiple embedding spaces to provide more critical feature discriminations.

## 5. Optimization method

The optimization-based few-shot learning method uses model-dependent external metrics to replace the model-agnostic method. The model-agnostic method uses a learning method based on stochastic gradient descent to define a common optimization method compatible with all models. The algorithm aims to optimize all potential classes, not just a specific data set.

### 5.1. Self-learning

Model-Agnostic Meta-Learning (MAML) (Finn et al., 2017) aims to optimize the parameters initialization to achieve fast learning. MAML uses the parameters of the subtask and then updates the parameters according to the direction of the second gradient update. The second-order update of each task is used to implemented each update of the base model. Compared to MAML, Reptile (Nichol et al., 2018) is a strategy that repeatedly samples a task, train on it, and shifts the initialization in the direction of the learned weights on that task. Latent Embedding Optimization (LEO) (Rusu et al., 2018) learns a low-dimensional latent embedding of model parameters and uses optimization-based meta-learning in this space. The issue of optimizing in high-dimensional spaces in extreme low-data regimes is resolved by learning low-dimensional latent representation.

### 5.2. Mutual learning

Knowledge distillation (Hinton et al., 2015) can not only be used for model compression but also improve the performance of a complex model through optimization strategies such as mutual learning and self-learning. Rajasegaran et al. (2020) propose a Knowledge Distillation method to promote the representation ability of deep neural networks for few-shot learning. Intra-class diversity is guaranteed by self-supervision and a knowledge distillation network secures inter-class discrimination through this method.

## 6. Model-based method

### 6.1. Memory-based

A series of model architectures incorporate an external memory component to advance their learning process. The two-dimensional matrix is the commom type of the external memory component which is also called the memory bank or memory matrix. The neural networks can access novel information and retrieve previously stored information to the memory serving as a storage buffer (Parnami and Lee, 2022). Note that this memory component is not the same as internal memory in RNN (Santoro et al., 2016a) or LSTMs (Ravi and Larochelle, 2017). In the context of few-shot learing, memory as an external component can alleviate the burden of learning in a low-data regime and enable more rapid generalization (Parnami and Lee, 2022). Santoro et al. (2016b) demonstrate the capability of an enhanced memory neural network to rapidly assimilate novel data samples, and use this data to conduct precise predictions based on just a few samples. They also introduce a new method to access an external memory that focuses on the content of the memory, instead of additionally using focusing mechanisms based on memory location. Cai et al. (2018) present Memory Matching Networks (MM-Net), a new deep architecture exploring the training process according to the philosophy that training and test conditions must correspond. Simultaneously, MM-Net can train a unified model regardless of the number and category of the data samples.

### 6.2. Rapid adaptation

The following model-based methods employ techniques such as “fast-weights” to rapidly adapt the parameters of a model for a given task (Parnami and Lee, 2022). Munkhdalai and Yu (2017) propose a new meta-learning method, Meta Networks (MetaNet), which learns a meta-level knowledge across tasks and displaces its inductive biases through rapid parameterization for fast generalization. They further present a mechanism through which artificial neural networks can learn fast adaptation (Munkhdalai et al., 2018), which can adapt on-the-fly, with limited data, to novel tasks called conditionally shifted neurons.

### 6.3. Multi-task learning

Multi-task Learning aims to learn several correlated tasks together. In the learning process, a shared representation is exploited to share and complement the learned domain-related information, facilitate mutual learning, and promote the effect of generalization. There are two ways to share representations in multi-task learning: parameter sharing and parameter tying. (1) Parameter Sharing is the strategy that directly shares some parameters among tasks. By learning separate embedding functions for both the source and target tasks, the original and generated samples are initially mapped to a task-specific space and then embedded *via* a shared variational autoencoder (Benaim and Wolf, 2018). (2) Parameter tying is the strategy that encourages parameters of different tasks to be similar. Luo et al. (2017) present two CNNs, one for the source task and the other for the target task. Layers of these two CNNs are aligned using some specially designed regularization terms.

## 7. Applications and challenges of few-shot learning

Few-shot learning can be used to learn a good model with a few examples. Few-shot learning is more suitable for practical applications. It can be applied for the following scenarios: (1) Classification of hyperspectral images (Xi et al., 2022), which is widely used in environmental monitoring, mineral exploration, military target recognition, etc. (2) Object detection (Han et al., 2022), which contains intrusion detection, endangered-animal detection, remote sensing image target detection, etc. (3) Robot (Kok et al., 2022), which can be trained to learn a movement by imitating a single demonstration or learning manipulation actions from a few demonstrations.

Few-shot learning has made some progress but also faces challenges: (1) Interpretability of few shot learning. The deep learning model has black-box nature. In few-shot transfer learning, it is difficult to learn what features are preserved during feature and parameter transfer, making it more challenging to tune parameters. Improving the interpretability of few-shot learning can help to find appropriate transfer features between the source and the target domain. (2) Enforced pre-trained model. In the existing few-shot learning methods, whether based on metric or optimization methods, it is necessary to pre-train the model on a large number of non-target datasets. Therefore, the pre training of the model still requires a lot of annotated data. To fundamentally solve the few samples problem, we can research methods that use other prior knowledge instead of the pre-trained model.

## 8. Conclusion and prospect

Few-shot learning is more difficult than traditional deep learning. However, it has more extensive and practical application values in the real-word. Few-shot learning can learn new tasks from prior knowledge, which reduces the model's dependence on data. In this mini review, we first clarify the definition of the few-shot learning problem. We then analyze the four categories of few-shot learning: data augmentation method, metric-based method, optimization method and model-based method. We compare the pros and cons of each category.

In the domain of machine learning, the scale and quality of datasets in different tasks are essential issues that limit the performance of machine learning systems. Few-shot learning aims to learn novel tasks with a small number of data. Thus, there are some prospects: (1) Enhanced evaluation of data distribution. Only a few data samples can be used to access the true data distribution for the few-shot learning. Therefore, the baseline dataset is completed and developed to assess the generalization capability of a model to fine-grained detail for few-shot learning, which will be practical and advanced. (2) More effective meta-knowledge learning from previous tasks. No interpretable theory has yet appeared to account for the causal correlation between tasks behind meta-learning. As the framework for causal theory evolves, the meta-learning framework would probably tend to become more general. (3) Making full use of multimodal information. There is an urgent demand to design a powerful pretrained model involving the fusion of three and more modalities for efficient feature reuse in multimodality, which can learn a few data sample tasks in the scenarios without supervised information and quickly shift to data from various domains.

## Author contributions

RZ wrote the manuscript with help from QL. QL assisted in way of writing. All authors contributed to the article and agreed to the submitted version.

## References

[B1] Bateni P. Barber J. van de Meent J.-W. Wood F. (2022). “Enhancing few-shot image classification with unlabelled examples,” in Proceedings of the IEEE/CVF Winter Conference on Applications of Computer Vision (Waikoloa, HI: IEEE), 2796–2805.

[B2] Benaim S. Wolf L. (2018). “One-shot unsupervised cross domain translation,” in Advances in Neural Information Processing Systems 31 (2108–2118).

[B3] Cai J. Zhang Y. Guo J. Zhao X. Lv J. Hu Y. (2022). St-pn: a spatial transformed prototypical network for few-shot sar image classification. Remote Sens. 14, 2019. 10.3390/rs14092019

[B4] Cai Q. Pan Y. Yao T. Yan C. Mei T. (2018). “Memory matching networks for one-shot image recognition,” in Proceedings of the IEEE Conference on Computer Vision and Pattern Recognition (Salt Lake City, UT: IEEE), 4080–4088.

[B5] Chen H. Li H. Li Y. Chen C. (2022). “Multi-level metric learning for few-shot image recognition,” in International Conference on Artificial Neural Networks (Springer), 243–254.

[B6] Chen Q. Chen Z. Luo W. (2022). Feature transformation for cross-domain few-shot remote sensing scene classification. arXiv preprint arXiv:2203.02270. 10.48550/arXiv.2203.02270

[B7] Chi W. Xiang Z. Guo F. (2022). Few-shot learning for deformable image registration in 4dct images. Br. J. Radiol. 95, 20210819. 10.1259/bjr.20210819 34662242 PMC8722248

[B8] Chikontwe P. Kim S. Park S. H. (2022). “Cad: co-adapting discriminative features for improved few-shot classification,” in Proceedings of the IEEE/CVF Conference on Computer Vision and Pattern Recognition, 14554–14563.

[B9] Ding C. Li Y. Wen Y. Zheng M. Zhang L. Wei W. Zhang Y. (2021). Boosting few-shot hyperspectral image classification using pseudo-label learning. Remote Sens. 13, 3539. 10.3390/rs13173539

[B10] Finn C. Abbeel P. Levine S. (2017). “Model-agnostic meta-learning for fast adaptation of deep networks,” in International Conference on Machine Learning (PMLR), 1126–1135.

[B11] Gao F. Cai L. Yang Z. Song S. Wu C. (2022). “Multi-distance metric network for few-shot learning,” in International Journal of Machine Learning and Cybernetics, 2495–2506.

[B12] Gao H. Xiao J. Yin Y. Liu T. Shi J. (2022). A mutually supervised graph attention network for few-shot segmentation: the perspective of fully utilizing limited samples. IEEE Trans. Neural Netw. Learn. Syst. 1–13. 10.1109/TNNLS.2022.3155486 35286269

[B13] Garcia V. Bruna J. (2017). Few-shot learning with graph neural networks. arXiv preprint arXiv:1711.04043. 10.48550/arXiv.1711.04043

[B14] Goodfellow I. Pouget-Abadie J. Mirza M. Xu B. Warde-Farley D. Ozair S.. (2020). Generative adversarial networks. Commun. ACM 63, 139–144. 10.1145/3422622

[B15] Han G. Ma J. Huang S. Chen L. Chang S. -F. (2022). “Few-shot object detection with fully cross-transformer,” in Proceedings of the IEEE/CVF Conference on Computer Vision and Pattern Recognition (IEEE), 5321–5330. Available online at: https://openaccess.thecvf.com/content/CVPR2022/papers/Han_Few-Shot_Object_Detection_With_Fully_Cross-Transformer_CVPR_2022_paper.pdf

[B16] Hinton G. Vinyals O. Dean J. (2015). Distilling the knowledge in a neural network. arXiv preprint arXiv:1503.02531 2(7). 10.48550/arXiv.1503.02531

[B17] Hou R. Chang H. Ma B. Shan S. Chen X. (2019). “Cross attention network for few-shot classification,” in Advances in Neural Information Processing Systems, Vol. 32, 4005–4016.

[B18] Huang H.-P. Sun D. Liu Y. Chu W.-S. Xiao T. Yuan J. Adam H. Yang M.-H. (2022). Adaptive transformers for robust few-shot cross-domain face anti-spoofing. arXiv preprint arXiv:2203.12175. 10.1007/978-3-031-19778-9_3

[B19] Huang J. Chen F. Wang K. Lin L. Zhang D. (2022). “Enhancing prototypical few-shot learning by leveraging the local-level strategy,” in ICASSP 2022-2022 IEEE International Conference on Acoustics, Speech and Signal Processing (ICASSP) (Singapore: IEEE), 1660–1664.

[B20] Jia X. Su Y. Zhao H. (2022). Few-shot learning *via* relation network based on coarse-grained granulation. Appl. Intell. 1–13. 10.1007/s10489-022-03332-7

[B21] Jian Y. Torresani L. (2022). Label hallucination for few-shot classification. Proc. AAAI Conf. Artif. Intell. 36, 7005–7014. 10.1609/aaai.v36i6.20659

[B22] Johnander J. Edstedt J. Felsberg M. Khan F. S. Danelljan M. (2022). “Dense gaussian processes for few-shot segmentation,” in European Conference on Computer Vision (Springer), 217–234.

[B23] Kok V. Olusanya M. Ezugwu A. (2022). A few-shot learning-based reward estimation for mapless navigation of mobile robots using a siamese convolutional neural network. Appl. Sci. 12, 5323. 10.3390/app12115323

[B24] Liu L. Hamilton W. Long G. Jiang J. Larochelle H. (2020). A universal representation transformer layer for few-shot image classification. arXiv preprint arXiv:2006.11702. 10.48550/arXiv.2006.11702

[B25] Luo Z. Zou Y. Hoffman J. Fei-Fei L. F. (2017). “Label efficient learning of transferable representations acrosss domains and tasks,” in Advances in Neural Information Processing Systems 30.

[B26] Munkhdalai T. Yu H. (2017). “Meta networks,” in International Conference on Machine Learning (PMLR), 2554–2563. PMC6519722 31106300

[B27] Munkhdalai T. Yuan X. Mehri S. Trischler A. (2018). “Rapid adaptation with conditionally shifted neurons,” in International Conference on Machine Learning (PMLR), 3664–3673.

[B28] Nichol A. Achiam J. Schulman J. (2018). On first-order meta-learning algorithms. arXiv preprint arXiv:1803.02999. 10.48550/arXiv.1803.0299933729779

[B29] Parnami A. Lee M. (2022). Learning from few examples: a summary of approaches to few-shot learning. arXiv preprint arXiv:2203.04291. 10.48550/arXiv.2203.04291

[B30] Rajasegaran J. Khan S. Hayat M. Khan F. S. Shah M. (2020). Self-supervised knowledge distillation for few-shot learning. arXiv preprint arXiv:2006.09785. 10.48550/arXiv.2006.0978533668138

[B31] Ravi S. Larochelle H. (2017). “Optimization as a model for few-shot learning,” in International Conference on Learning Representations.

[B32] Rusu A. A. Rao D. Sygnowski J. Vinyals O. Pascanu R. Osindero S.. (2018). Meta-learning with latent embedding optimization. arXiv preprint arXiv:1807.05960. 10.48550/arXiv.1807.05960

[B33] Santoro A. Bartunov S. Botvinick M. Wierstra D. Lillicrap T. (2016a). “Meta-learning with memory-augmented neural networks,” in International Conference on Machine Learning (PMLR), 1842–1850.

[B34] Santoro A. Bartunov S. Botvinick M. Wierstra D. Lillicrap T. (2016b). One-shot learning with memory-augmented neural networks. arXiv. 10.48550/arXiv.1605.06065

[B35] Shen Y. Shuai X. (2022). “Meta-learning fine-tuned feature extractor for few-shot image classification: a case study on fine-tuning cnn backbone with transformer for few-shot learning,” in 2022 4th Asia Pacific Information Technology Conference, 9–14.

[B36] Singh P. Mazumder P. (2022). Dual class representation learning for few-shot image classification. Knowl. Based Sys. 238, 107840. 10.1016/j.knosys.2021.107840

[B37] Snell J. Swersky K. Zemel R. (2017). “Prototypical networks for few-shot learning,” in Advances in Neural Information Processing Systems, Vol. 30, 4080–4090.34495842

[B38] Su Y. Zhao H. Lin Y. (2022). Few-shot learning based on hierarchical classification *via* multi-granularity relation networks. Int. J. Approximate Reason. 142, 417–429. 10.1016/j.ijar.2021.12.013

[B39] Subedi B. Sathishkumar V. Maheshwari V. Kumar M. S. Jayagopal P. Allayear S. M. (2022). Feature learning-based generative adversarial network data augmentation for class-based few-shot learning. Math. Problems Eng. 2022, 9710667. 10.1155/2022/9710667

[B40] Sung F. Yang Y. Zhang L. Xiang T. Torr P. H. Hospedales T. M. (2018). “Learning to compare: relation network for few-shot learning,” in Proceedings of the IEEE Conference on Computer Vision and Pattern Recognition (Salt Lake City, UT: IEEE), 1199–1208.

[B41] Vanschoren J. (2019). “Meta-learning,” in Automated Machine Learning (Cham: Springer), 35–61

[B42] Wang Y. Yao Q. Kwok J. T. Ni L. M. (2020). Generalizing from a few examples: a survey on few-shot learning. ACM Comput. Surveys 53, 1–34. 10.1145/3386252

[B43] Xi B. Li J. Li Y. Song R. Hong D. Chanussot J. (2022). “Few-shot learning with class covariance metric for hyperspectral image classification,” in IEEE Transactions on Image Processing, Vol. 31 (IEEE), 5079–092. 10.1109/TIP.2022.3192712 35881603

[B44] Yang Z. (2022). Task-prior conditional variational auto-encoder for few-shot image classification. arXiv preprint arXiv:2205.15014. 10.48550/arXiv.2205.15014

[B45] Zhang M. Zhang J. Lu Z. Xiang T. Ding M. Huang S. (2021). “Iept: instance-level and episode-level pretext tasks for few-shot learning,” in International Conference on Learning Representations, 1–16.

[B46] Zhang Y. Tang H. Jia K. (2018). “Fine-grained visual categorization using meta-learning optimization with sample selection of auxiliary data,” in Proceedings of the European Conference on Computer Vision (ECCV), 233–248.

[B47] Zhao Z. Liu Q. Cao W. Lian D. He Z. (2022). Self-guided information for few-shot classification. Pattern Recognit. 131, 108880. 10.1016/j.patcog.2022.108880

